# Perceived Job Insecurity and Depressive Symptoms among Italian Dentists: The Moderating Role of Fear of COVID-19

**DOI:** 10.3390/ijerph17155338

**Published:** 2020-07-24

**Authors:** Roberta Gasparro, Cristiano Scandurra, Nelson Mauro Maldonato, Pasquale Dolce, Vincenzo Bochicchio, Alessandra Valletta, Gilberto Sammartino, Pasquale Sammartino, Mauro Mariniello, Alessandro Espedito di Lauro, Gaetano Marenzi

**Affiliations:** 1 Department of Neuroscience, Reproductive Sciences and Dentistry, University of Naples Federico II, 80131 Naples, Italy; roberta.gasparro@unina.it (R.G.); cristiano.scandurra@unina.it (C.S.); nelsonmauro.maldonato@unina.it (N.M.M.); alessandra.valletta@unina.it (A.V.); alessandroespedito.dilauro@unina.it (A.E.d.L.); gaetano.marenzi@gmail.com (G.M.);; 2 Department of Public Health, University of Naples Federico II, 80131 Naples, Italy; pasquale.dolce@unina.it; 3 Department of Humanistic Studies, University of Calabria, 87036 Arcavacata di Rende, Italy; vincenzo.bochicchio@unical.it; 4 Private Practice, 80131 Naples, Italy; pasqualesammartino91@gmail.com (P.S.); mauro.mariniello@gmail.com (M.M.)

**Keywords:** COVID-19, dentists, fear, job insecurity, depression

## Abstract

Containment measures adopted to reduce the spread of coronavirus disease 2019 (COVID-19) have produced a general perception of job insecurity. Dentists have been highly affected by such measures, as they represent an easy source of contagion. As perceived job insecurity is associated with psychological distress and Italian dentists have been highly affected by the COVID-19 outbreak in terms of potential financial loss and the risk of being infected, this study aimed at assessing whether the fear of COVID-19 moderated the effect of perceived job insecurity on depressive symptoms. This cross-sectional online study has included 735 Italian dentists recruited during the lockdown and ranging in age from 27 to 70 years old (495 men and 240 women). A quantile regression model with an inference based on the median and with an interaction term between the fear of COVID-19 and perceived job insecurity has been used to estimate the hypothesized associations. The results indicated that both perceived job insecurity and fear of COVID-19 were positively associated with depressive symptoms, and that the effect of perceived job insecurity on depressive symptoms was weaker among those with a low fear of COVID-19. The findings may inform public health policies for dentists in relation to reducing the risk of developing negative mental health outcomes.

## 1. Introduction

On January 8, 2020, a novel coronavirus was officially announced as the causative pathogen of coronavirus disease 2019 (COVID-19) by the Chinese Center for Disease Control and Prevention [1]. The COVID-19 epidemic started from Wuhan, Hubei province, in China, last December and, within a few months, it has spread globally [2]. On 11th March 2020, the World Health Organization (WHO) declared it as a pandemic disease [3]. The novel coronavirus was initially named 2019-nCoV and officially as severe acute respiratory syndrome coronavirus 2 (SARSCoV-2). The common transmission routes of this novel coronavirus include direct transmission (coughing, sneezing, and droplet inhalation transmission) and contact transmission (contact with oral, nasal, and eye mucous membranes) [4].

On account of the high transmissibility of the 2019-nCoV and to the evidence that it may be airborne through aerosols formed during medical procedures [5], healthcare professionals are exposed to a higher risk of getting infected due to their close contact with infected patients. Among different healthcare professionals, dentists are particularly affected by the risk of being infected by COVID-19, as they not only work in close contact with patients but are also exposed to aerosols and droplets splashing out of the patient’s oral cavity [6]. Thus, dentists are also at higher risk of infecting their patients. For these reasons, severe restrictions have been adopted by governments to avoid this source of contagion. In Italy, which is the context of the current study, Italian dentists have been allowed to practice only emergency/urgent procedures during the whole period of the lockdown. Specifically, dental professionals have been urged by the government to suspend all non-urgent appointments to limit the movement of people. This order has meant that all dental hygienists have effectively been suspended from work while many dentists have been left uncertain as to what exactly constitutes emergency care. The Italian Society of Periodontology and Implantology (SIdP) recommends that patients only access dental care if their appointments cannot be deferred. Some examples of dental emergencies of an urgent nature, as identified by the SIdP, include acute pain, infections such as a gingival abscess, and trauma. However, if the patient is at greater risk of contracting SARS-CoV-2, shows symptoms or signs attributable to the SARS-CoV-2 infection, or is in quarantine, the treatment should be postponed to a later time.

Containment measures (i.e., social distancing, self-isolation, travel restrictions, etc.) adopted to reduce the spread of COVID-19 have resulted in a reduced workforce across many economic sectors [7], as well as a general perception of job insecurity [8], namely the fearful of job loss or worry about job continuation [9]. Perceived job insecurity has been found to be a fundamental stressor during the COVID-19 outbreak [10], as it has a negative impact on the individual’s financial capacity due to the high risk of financial loss [11]. In relation to dentists, a survey conducted by the Irish Dental Association with 369 dentists reported that one-fifth of participants closed their practices temporarily or permanently, and that around three-quarters of the sample expected a financial loss of over 70% amid the COVID-19 outbreak [12]. Similarly, in an Italian survey of 356 dentists, exploring the practical and emotional consequences of the COVID-19 outbreak on daily dental clinical practice, it was found that all the participants had closed their practices or had limited their activity only to urgent procedures and that 92.7% of patients had canceled their previously made appointments [13]. Such data make it plausible to hypothesize that dentists have experienced high levels of perceived job insecurity during the COVID-19 outbreak.

Previous studies have highlighted that perceived job insecurity has serious consequences not only on the individuals’ financial capacity, but also on their mental health [14,15,16,17,18]. For instance, Zhang et al. [19] found that people who have been forced to stop working due to the COVID-19 outbreak reported an increase in distress and a decline in health. Similarly, Mihashi et al. [20] reported that, during the severe acute respiratory syndrome (SARS) outbreak of 2003, income reduction was a significant predictor of psychological disorders. Thus, it seems plausible to hypothesize that, as Italian dentists have been the category of health-care professionals most significantly affected economically by the containment measures adopted in Italy [13] and worldwide [21,22] during the lockdown, they are at risk of experiencing greater levels of perceived job insecurity and, as a consequence, mental health problems. Accordingly, although not directly associated with the perceived job insecurity, some studies have found a considerable degree of psychological distress among dentists during the COVID-19 outbreak [13,23].

Additionally, previous studies have highlighted that, despite the severe restrictions implemented by governments worldwide, dentists might continue to be worried about being infected by the virus. For instance, Consolo et al. [13] found that 85% of their sample of dentists (N = 356) were anxious about contracting COVID-19 during their clinical activity. Similarly, Ahmed et al. [24] found that 87% of 669 dentists from 30 different countries were afraid of getting infected with COVID-19. A fear of contracting COVID-19 has been found to be associated with negative mental health outcomes. For instance, in a study conducted with 338 Israeli dentists and dental hygienists, an elevated level of psychological distress was found in those with a higher fear of contracting COVID-19 [23]. However, no previous studies have explored the potential interactions between the fear of being infected by COVID-19 and perceived job insecurity either in the general population or in the profession of dentists. Indeed, it is possible to hypothesize that the effect of perceived job insecurity on depressive symptoms increases with respect to dentists experiencing a higher level of fear of being infected with COVID-19 compared to those experiencing lower levels of fear. It is likely, in fact, that dentists more afraid of the possibility of being infected by COVID-19 would consider their job prospects more at risk than those less frightened because, if they were to be infected, they would no longer be able to work for a certain period. This could mean that what was previously experienced as a perception of job insecurity would become a real economic loss and, accordingly, the greater the job insecurity the greater the probability of developing adverse mental health outcomes [25,26]. Therefore, it seems plausible to hypothesize that the perception of job insecurity interacts with the fear of being infected in relation to the development of depressive symptoms.

Therefore, as perceived job insecurity has proved to be consistently associated with psychological distress [14,15,16,17,18] and Italian dentists have been significantly affected by the COVID-19 outbreak in terms of potential financial loss [12,13] and risk of being infected [24], we were interested in assessing the associations between perceived job insecurity, the fear of COVID-19, and mental health (i.e., depressive symptoms). Specifically, we hypothesized that perceived job insecurity and the fear of COVID-19 would be positively associated with depressive symptoms and that the fear of COVID-19 would moderate the relationship between perceived job insecurity and depressive symptoms. Specifically, we hypothesized that the association between perceived job insecurity and depressive symptoms would be weaker among those with a lower fear of COVID-19 compared to their counterparts who had a higher-fear.

**Figure 1:**
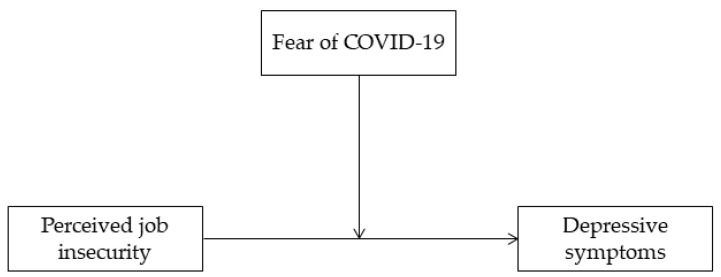
The Moderation Model Hypothesized.

The moderation model hypothesized is depicted in Figure 1.

## 2. Materials and Methods

### 2.1. Procedures

In this study we have used a cross-sectional online survey distributed among Italian dentists, contacting both Italian stakeholders in the field of dentistry and representatives of the National Order of Dentists, who were requested to share the survey among their members. By clicking on the link provided, the participants were first directed to the informed consent in relation to the study, where they were able to read objectives, benefits and risks of participation, and information about the researchers. They were informed about the anonymity of the survey, as well as about their right to terminate their involvement at any moment they desired. Their privacy was guaranteed in accordance with the EU General Data Protection Regulation 2016/679. The study was designed in accordance with the Declaration of Helsinki and was approved by the Ethical Committee of the University of Naples Federico II (protocol number: 159/20; date: 23/04/2020). The study was also conducted in accordance with the “Strengthening the Reporting of Observational Studies in Epidemiology” (STROBE) statement for cross-sectional studies.

### 2.2. Participants

The participants were recruited in Italy during the national lockdown, specifically between 17th April and 3rd May 2020. The inclusion criteria were: (1) being a Doctor of Dental Science; (2) having at least 2 years of professional experience; (3) working in Italy; and (4) not being retired. We decided to include dentists with at least 2 years of dental practice as a minimum period of employment was considered as necessary to explore the dimension of perceived job insecurity during the COVID-19 outbreak. Indeed, as we were interested in assessing such job insecurity, it was necessary to ensure that the participants would be able to compare their current work situation with that before the COVID-19 outbreak. A total of 820 Italian dentists, out of about 50,000 listed on the national register, completed the survey. Among these, 85 did not satisfy one or more of the inclusion criteria. Thus, the final sample was composed of 735 participants. The socio-demographic characteristics of the sample are reported in Table 1.

### 2.3. Measures

#### 2.3.1. Socio-Demographic Characteristics

The socio-demographic variables assessed in the current study included age, gender (men vs. women), type of job (private practice without colleagues, private practice with colleagues, hospital facility, and/or private clinic), field of dentistry field (e.g., general dentistry, orthodontics, etc.), and personal knowledge of people who had died due to COVID-19 and the number of such people known personally. 

#### 2.3.2. Perceived Job Insecurity

Perceived job insecurity was measured through a single-item based on the work by Strazdins et al. [27]. This item assessed the fear of job loss, which is a core dimension of perceived job insecurity, and was adapted to relate to the COVID-19 outbreak, as follows: “How secure do you feel about your job or career prospects in your current workplace due to the COVID-19 outbreak?” The response options ranged from “very secure” to “not at all secure”, on a 5-point Likert scale, with higher scores indicating a higher perceived job insecurity. 

#### 2.3.3. Fear of COVID-19

The fear of COVID-19 was measured through the Fear of COVID-19 Scale (FCV-19S by Ahorsu et al. [28]; Italian version by Soraci et al. [29]), a 7-item scale assessing the fear of being infected by COVID-19. The responses ranged from 1 (“strongly disagree”) to 5 (“strongly agree”), with higher scores reflecting higher levels of contracting COVID-19. Scores ranged from 7 to 35. An example item is “It makes me uncomfortable to think about coronavirus-19”. The Cronbach’s alpha for the current sample was 0.87.

#### 2.3.4. Depressive Symptoms

The presence of depressive symptoms was assessed through the short version of the DSM-5 Severity Measure for Depression–Adult (SMDA; Spitzer et al. [30]; Italian version by Fossati et al. [31]), a 9-item scale measuring the severity of depressive symptoms over the last 7 days on a 4-point Likert scale, ranging from “not at all” to “nearly every day.” The initial question is “Over the last 7 days, how often have you been bothered by any of the following problems?” An example item is “Feeling tired or having little energy.” The total score may range from 0 to 27, with higher scores indicating a greater severity of depressive symptoms. The Cronbach’s alpha for the current sample was 0.90.

### 2.4. Statistical Analyses

All statistical analyses were performed using SPSS and the R software for statistical computing. As the data had a skewed distribution, the Spearman’s coefficient was used as a measure of bivariate correlations between the main variables of the study. As the socio-demographic variables may influence the perceived job insecurity, fear of COVID-19, and depressive symptoms, we adjusted the successive models to take into account potential confounding variables, including gender (men vs. women), age, years of dental practice, personal knowledge of people who had died due to COVID-19 (yes vs. no), and number of such people known personally. Most of the participants worked both in the public and private sector, as the Italian public health system allows health-care professionals to work in more than one sector; therefore, it was not possible to differentiate the sample into subgroups. The relationships between all the socio-demographic quantitative variables and the main variables of the current study were tested through the Spearman’s correlation coefficient. On account of the categorical nature of the items gender and knowledge of people who had died due to COVID-19, we used the Mann–Whitney U test to test for significant differences of the main variable distributions between groups. The relationships of all the other socio-demographic variables with the main variables of the current study were tested through the Spearman’s correlation coefficient. In the final model, only covariates showing a significant difference based on Mann–Whitney U test or significant correlations with the main variables of the study were included (i.e., age, gender, and number of people who had died due to COVID-19 known personally). However, as years of professional experience and age were highly correlated (r = 0.92; *p* < 0.001), only the age of participants was included in the final model to avoid possible problems of multicollinearity.

As the distribution of the depressive symptoms scale (i.e., the dependent variable) was highly skewed, a quantile regression model with an inference based on the median was used to estimate the effect of perceived job insecurity on depressive symptoms. Furthermore, in order to test for the moderating effect of the fear of COVID-19 in the relationship between perceived job insecurity and depressive symptoms, we included in the model an interaction term between the fear of COVID-19 and perceived job insecurity. The effect of perceived job insecurity on depressive symptoms was then estimated for three levels of the fear of COVID-19 (Q_1_, Median, and Q_3_) and the regression lines obtained were plotted. The 95% confidence intervals (CI) were provided for the parameters. The level of significance for all tests was set as *p* < 0.05.

## 3. Results

The means, standard deviations, and bivariate correlations between perceived job insecurity, fear of COVID-19, and depressive symptoms are shown in Table 2. The results highlighted a positive correlation between perceived job insecurity, fear of COVID-19, and depressive symptoms.

With regard to the socio-demographic factors considered as potential confounding variables, both age (r = −0.12; *p* = 0.001) and years of professional experience (r = −0.12; *p* = 0.001) were negatively correlated only with depressive symptoms. In comparison with the males, the female dentists demonstrated higher levels of fear of COVID-19 (Mdn_men_ = 2, Mdn_women_ = 2.28; U = 49,062; *p* < 0.001) and depressive symptoms (Mdn_men_ = 0.44, Mdn_women_ = 0.55; U = 53,793.500; *p* = 0.037), but similar levels of job insecurity. A personal knowledge of people who had died due to COVID-19 did not show any significant differences on the variables, while the number of people who had died due to COVID-19 known personally positively correlated only with depressive symptoms (r = 0.14; *p* = 0.026). 

The results of the quantile regression analysis including only the main effects indicated that both perceived job insecurity (b = 0.58, *p* < 0.001, 95% CI [0.35,0.70]) and fear of COVID-19 (b = 2.11, *p* < 0.001, 95% CI [1.58,2.60]) were positively associated with depressive symptoms, confirming our first hypothesis. The model with the interaction term showed that the fear of COVID-19 significantly moderated the relationship between perceived job insecurity and depressive symptoms (b = 0.61, *p* = 0.007, 95% CI [0.14,1.06]), suggesting that the association between perceived job insecurity and depressive symptoms increases with the levels of fear of COVID-19, confirming our second hypothesis. The regression coefficients of age and number of people who had died known personally were also statistically significant (*p* = 0.014 and *p* = 0.049, respectively).

**Figure 2:**
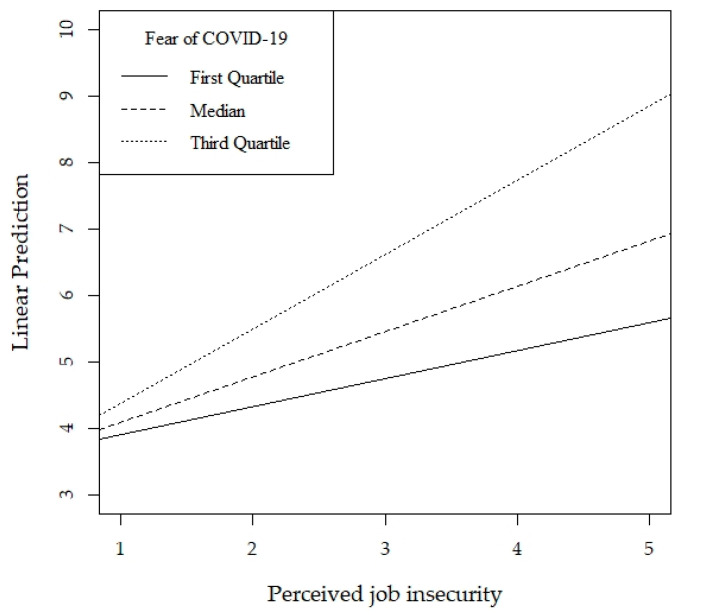
Interaction Effect of Perceived Job Insecurity by Fear of COVID-19 on Depressive Symptoms.

As is shown in a graphical form in Figure 2, the effect of perceived job insecurity on depressive symptoms is weaker among those with a low fear of COVID-19 compared to those with a higher-fear.

## 4. Discussion

The present study examined whether the fear of COVID-19 moderated the relationship between perceived job insecurity and depressive symptoms among Italian dentists during the COVID-19 outbreak. Our results supported the moderation effect expected, showing that the association between perceived job insecurity and depressive symptoms was weaker in those with low fear of COVID-19 compared to those with a higher fear. To the best of our knowledge, this is the first study assessing the moderating role of fear of COVID-19 on the relationship between perceived job insecurity and mental health in Italian dentists. Such a result may inform public health policies to the dentistry profession during a pandemic.

With regard to the descriptive analyses, the results of the current study highlighted three different associations. These results have to be read in the context of the COVID-19 outbreak, as a potential depiction of the subgroups of dentists who may experience greater psychological risks. Accordingly, the first result highlighted that being younger and having fewer years of dental practice was associated with higher levels of depressive symptoms. This finding is similar to that by Huang and Zhao [32], who found that, among Chinese participants, younger people were more likely to develop anxiety and depressive symptoms during the COVID-19 outbreak than their older counterparts. Similarly, in assessing levels of anxiety and depression in Chinese medical staff working in COVID-19 units, Liang et al. [33] found that younger medical staff showed higher rates of depression. Thus, these studies seem to highlight that a younger age may represent a psychological risk factor increasing the likelihood of developing depressive symptoms during epidemics, in contrast to an older age, which is an objective risk factor for a greater severity and mortality [34]. This finding may be due, as during the SARS outbreak, to differences in coping with the psychological challenges associated with the outbreak, with younger individuals using less functional coping strategies [35]. Along the same lines, during the SARS outbreak, it was found that older people were more likely to take precautionary measures against the infection, and this proved to be associated with a lower psychological impact of the COVID-19 outbreak and lower levels of stress and depressive symptoms [36]. However, alternative explanations could be that younger or shorter-tenured dentists may have a greater fear of job loss due to their shorter employment history, may be in a more insecure financial condition and may be more greatly concerned about their career development than their older and more experienced counterparts. The second result indicated that the female dentists seemed to have a greater fear of COVID-19 and depressive symptoms than males. This finding is in line with the results obtained by Wang et al. [37] who found that women suffered a greater psychological impact of the COVID-19 outbreak, confirming epidemiological studies which report that women are at higher risk of depressive symptoms than men [38]. Finally, the third result indicated that knowing more than one people who had died due to COVID-19 was associated with higher levels of depressive symptoms. This finding is in line with a previous work performed during the SARS outbreak, in which people knowing someone who had SARS were more likely to report psychological distress [36], thus representing a risk factor increasing the fear of infection. Furthermore, knowing several people who had died due to COVID-19 would be likely to provoke feelings of grief and mourning, thus increasing depressive symptoms.

Regarding our main hypotheses, we found support for the association between perceived job insecurity and depressive symptoms, as well as for the moderating role of a fear of being infected by COVID-19 in this relationship. The relationship between perceived job insecurity and negative mental health outcomes is well established in literature [14,15,16,17,18] and might be explained by virtue of the fact that pandemics (e.g., the COVID-19 outbreak) may produce a great uncertainty about the future [39] and even about one’s own job, thus causing a temporary economic uncertainty. Indeed, although not directly assessed in the current study, it is plausible to hypothesize that many factors (e.g., the severity of the lockdown measures, the severe containment measures adopted by governments to reduce the risk of contagion within healthcare contexts, the regional economic conditions, the perceptions of alternative work, or the client portfolio of the dentists) may have affected the dentists’ perception of job insecurity, creating a strong economic uncertainty. As a consequence, such an economic uncertainty, together with the uncertainty about one’s own job and the future in general, may have produced in turn severe psychopathological conditions, such as depressive symptoms [39]. Based on our results, this seems particularly significant with respect to older workers, who have a lower level of perceived occupational mobility and a greater sensitivity to economic insecurity than their younger counterparts [14], and with respect to those who have a personal knowledge of people who have died due to COVID-19, as mourning may exacerbate depressive feelings. For instance, in a study presenting a mental health first aid service addressed to the general Italian population and health-care providers during the COVID-19 outbreak, Maldonato et al. [40] found that health-care professionals seemed to be less optimistic about their future quality of life than the general population, representing one of the most at risk groups in terms of psychological health. However, our findings showed that low levels of fear of contracting COVID-19 might ameliorate the negative effect that perceived job insecurity may have on depressive symptoms in dentists. As reported above, dentists work in close contact with patients while being exposed to aerosols and droplets splashing out of the patient’s oral cavity [6], and thus they may experience greater levels of fear of being infected by COVID-19. Fear represents an adaptive strategy serving the purpose of protecting oneself against potentially dangerous events. Nevertheless, when fear is disproportionate it may become maladaptive and harmful and can be a component of mental health problems [41]. Our findings suggest that this would be a plausible supposition also in respect of dentists, as a high fear of COVID-19 increases the strength of the effect of perceived job insecurity on mental health problems. Indeed, the COVID-19 outbreak has represented a social situation in which the fear of being infected has dramatically increased levels of negative mental health outcomes [40,42]. Indeed, the fear of COVID-19 has even exacerbated preexisting stress due to, among other factors, the loss of income and the economic crisis [43,44]. Moreover, previous studies [43,44] seem to have already highlighted potential associations between job insecurity, fear of COVID-19, and negative mental health outcomes, but none of these has explored such associations in detail. Considering the severe restrictions under which dentists have been obliged to work, it seems plausible to assert that being infected by COVID-19 could result in a temporary closing of their activity, thus increasing a real economic loss and exacerbating the levels of job insecurity. Furthermore, if we want to continue to consider the perception rather than the reality of job insecurity or loss, according to the social identity theory, people who lose a job, are in quarantine, or are unable to do their job for different reasons (for example, when the government prevents them from doing so because of public health issues, such as during the COVID-19 outbreak in Italy), may lack the necessary social confirmation, as well as the psychological and social support and verification, which they usually receive in their workplace thanks to their professional identity [45]. In this scenario, if the necessity for affiliation is high, the psychological distress during the crisis may increase, regardless of any actual job loss.

This study is innovative as it indicates potential psychological paths among Italian dentists leading to perceived job insecurity and so increasing the risk of developing depressive symptoms. However, although the sample was large, it was not representative of the category of Italian dentists. Furthermore, since the sample was recruited in a specific context (i.e., Italy), our results should be read as culturally contextualized, limiting the possibility of generalizing them to the general profession of dentists. Indeed, it is plausible to hypothesize that in other countries in which the restrictive measures were less severe than those realized in Italy, or in which the government implemented specific economic protective measures aimed at the dental profession, the findings may be different. Thus, future studies should consider replicating our study in other contexts.

Beyond these cultural considerations, our findings should be read in light of other important limitations. First, the study used a cross-sectional design which did not allow the drawing of conclusive inferences about the temporality and causality of the relationships between variables. Secondly, the sample was recruited during a specific phase of the outbreak (i.e., the lockdown). Both of these limitations should lead researchers to implement longitudinal studies to discern the cause-effect relationships between variables and to assess if fear of COVID-19 continues to act as a moderator in the relationship between perceived job insecurity and negative mental health outcomes even after the lockdown. Thirdly, perceived job insecurity was measured through a single-item question. Future studies should consider administering more composite measures, such as the Job Insecurity Scale [46]. Finally, as most of the participants in the current study worked both in private and public contexts, it was not possible to assess if this fundamental variable may increase the levels of perceived job insecurity. Future studies should consider recruiting samples of dentists working only in the private sector and others working only in the public sector.

### Implications for Public Health Policies

Despite these limitations, the findings of the current study might have significant implications for public health policies. Indeed, our results seem to indicate that intervening on high levels of perceived job insecurity or fear of COVID-19 may have a potentially positive effect on dentists’ mental health. For instance, as the fear of being infected by COVID-19 seems to exacerbate the effect that perceived job insecurity has on depressive symptoms, governments should allocate sufficient funds to provide greater quantities of, and more secure, personal protective equipment for dentists. This may alleviate potential financial losses and, at the same time, reduce the fear of being infected. To this end, the governments of some high-income countries (e.g., USA, Canada, and UK) and dental regulatory bodies have offered economic support to dental practices, addressing the financial needs of dentists and alleviating the potential negative effects of financial losses on health and security. In Italy, funds to overcome the financial loss of dentists have been allocated, but the amount that every professional could receive was negligible and allocated very late.

On the other hand, as even patients are afraid of undergoing dental procedures due to the high risk of being infected by dentists, other funds should be allocated to create awareness campaigns informing patients about the competence of dentists in fighting against emerging life-threatening diseases [22] and managing occupational health issues, such as hepatitis B and C, HIV, and cross-infection risks, which are all viruses more resistant than Sars-Cov2. This would increase the mutual trust [47,48,49,50,51], reducing the patients’ fear of being infected by their dentists and increasing the perception of dental settings as secure contexts. As a consequence, this might in turn decrease the perception of job insecurity in dentists and also its effect on mental health.

## 5. Conclusions

The current study has highlighted that the fear of COVID-19 would interact with perceived job insecurity in affecting depressive symptoms in Italian dentists. Thus, public health policies intervening on both the fear of COVID-19 and the perceived job insecurity may decrease the likelihood of the development of negative mental health outcomes. We hope that our findings will stimulate the Italian government to lighten the bureaucratic procedures and to allocate more funds for dentists and all professional categories at risk of job loss or financial problems during pandemics.

## Figures and Tables

**Table 1:** Socio-Demographic Characteristics of Participants (*N* = 735).

Variable	Participants N (%) or M ± SD
Age	
Mean	44.80 ± 12.44
Range	27–70
Gender	
Male	495 (67.3)
Female	240 (32.7)
Type of job *	
Private practice without colleagues	283 (38.5)
Private practice with colleagues	487 (66.3)
Hospital facility	116 (15.8)
Private clinic	50 (6.8)
Years of practice	17.81 ± 11.60
Specialization *	
General Dentistry	468 (63.7)
Orthodontics	171 (23.3)
Surgery and Implantology	303 (41.2)
Prosthetics	219 (29.8)
Periodontology	145 (19.7)
Conservative endodontics	288 (39.2)
Pedodontics	123 (16.7)
Personal knowledge of people who had died due to COVID-19	
Yes	236 (32.1)
No	499 (67.9)
Number of people who had died due to COVID-19 known personally	
1	197 (26.8)
2	28 (3.8)
3	5 (0.7)
4	4 (0.5)

Note: M = Mean; SD = Standard Deviation. * Most participants worked in more than one setting and had attained more than one specialization.

**Table 2:** Correlations Between Perceived Job Insecurity, Fear of COVID-19, and Depressive Symptoms.

Variables	1	2	3	Mdn (IQR)	M (SD)	Range
1. Perceived job insecurity	-			4 (3; 5)	3.57 (1.15)	1–5
2. Fear of COVID-19	0.44 ***	-		2 (1.57; 2.71)	15.03 (5.45)	7–35
3. Depressive symptoms	0.27 ***	0.41 ***	-	4 (2; 8)	5.66 (5.22)	0–27

*** *p* < 0.001. Mdn = Median; IQR = Interquartile Range; M = Mean; SD = Standard Deviation.
